# Evaluating the Chorioretinal Microcirculation in Preeclampsia with OCT-Angiography: A Narrative Literature Review

**DOI:** 10.3390/jcm14113913

**Published:** 2025-06-02

**Authors:** Evita Evangelia Christou, Ariel Yuhan Ong, Charlotte Frise, Assad Jalil, Tsveta Ivanova, Ilias Georgalas, Samantha R. de Silva

**Affiliations:** 1 Oxford Eye Hospital, Oxford University Hospitals NHS Foundation Trust, Oxford OX3 9DU, UK; 2 Manchester University NHS Foundation Trust, Manchester M13 9WL, UK; 3 Moorfields Eye Hospital NHS Foundation Trust, London EC1V 2PD, UK; 4 Institute of Ophthalmology, University College London, London WC1E 6BT, UK; 5 Queen Charlotte’s and Chelsea Hospital, Imperial College Healthcare NHS Trust, London W12 0HS, UK; 6 First Department of Ophthalmology, General Hospital G. Gennimatas, National and Kapodistrian University of Athens, 115 27 Athens, Greece; 7 Nuffield Department of Clinical Neurosciences, University of Oxford, Oxford OX3 9DU, UK

**Keywords:** macular microcirculation, peripapillary capillary plexus, choriocapillaris, optical coherence tomography angiography, preeclampsia, hypertensive disorder in pregnancy

## Abstract

**Background/Objectives:** The retinal microvasculature may reflect systemic vascular health and can be non-invasively imaged using optical coherence tomography angiography (OCTA). Investigation of the capillary plexuses in the macula and the peripapillary area could potentially provide insights into the pathophysiology of ocular manifestations in preeclampsia. We aimed to review the literature on OCTA metrics in preeclampsia to evaluate its use in this condition. **Methods:** A literature search was performed using the PubMed database, and studies published up to December 2024 were included. **Results:** We summarized the current evidence on chorioretinal microvascular changes in pregnancy and the ocular manifestations of preeclampsia. We reported findings from seven published studies characterizing the chorioretinal capillary plexuses in preeclampsia using OCTA. These revealed changes in microvasculature characteristics, such as foveal avascular zone size and vessel density in the macula and the peripapillary area; however, there was variability in reported parameters. **Conclusions:** Microvascular changes in the chorioretinal capillary plexus in preeclampsia were reported by several studies; however, results were inconsistent and may have been affected by multiple factors. Nevertheless, OCTA may have diagnostic and prognostic value, by providing evidence of microcirculation sequalae and aiding our understanding of ocular manifestations in this condition. Further studies are warranted to establish appropriate OCTA acquisition protocols and metrics, and whether these could guide clinical practice in preeclampsia.

## 1. Introduction

Preeclampsia is a hypertensive syndrome of pregnancy that usually occurs after 20 weeks of gestation. Diagnostic criteria include hypertension (>140/90 mmHg) and one of the following parameters: proteinuria (>0.3 g in 24 h), organ dysfunction or fetal growth restriction (ISSHP guidelines, 2019). Preeclampsia occurs in 3–7% of pregnancies, and ocular sequelae have been reported in one-third of patients [1,2]. Pathophysiological processes in preeclampsia may have a direct and profound impact on the retinal and choroidal microcirculation due to vasospasm, ischemia and increased vascular permeability, primarily leading to preeclampsia-related retinopathy, resembling that of hypertensive retinopathy. Therefore, the assessment of chorioretinal vasculature may detect blood flow changes that might contribute to our understanding of these ocular manifestations [1,2,3,4,5].

Advances in retinal imaging modalities have enabled direct visualization of the ocular microcirculation, giving new insights into the pathophysiology of various vascular conditions. There has been hesitancy about the use of interventional diagnostic procedures with intravenous dyes such as fluorescein angiography (FA) and indocyanine green angiography (ICGA) during pregnancy due to uncertainty regarding their effects on the developing fetus [1,2,3,4]. The advent of optical coherence tomography angiography (OCTA) has facilitated the non-invasive assessment of the microvascular network, providing a cross-sectional angiographic representation of the retinal and choroidal vasculature. OCTA may therefore be useful in assessing chorioretinal manifestations in preeclampsia [6,7,8,9,10].

To date, few studies have analyzed the OCTA characteristics of the retinal and choroidal capillary network in women with preeclampsia. Published reports have examined blood flow changes at the macula and the optic nerve in an attempt to clarify microvascular alterations in this condition [11,12,13,14,15,16,17]. In this review, we summarize the retinal vasculature changes in pregnancy, ocular manifestations of preeclampsia, evidence on OCTA characteristics in pregnancy and the effect of preeclampsia on the chorioretinal microvasculature. We also discuss possible explanations for the resultant ocular pathology and potential directions for further research.

## 2. Materials and Methods

**Figure 1:**
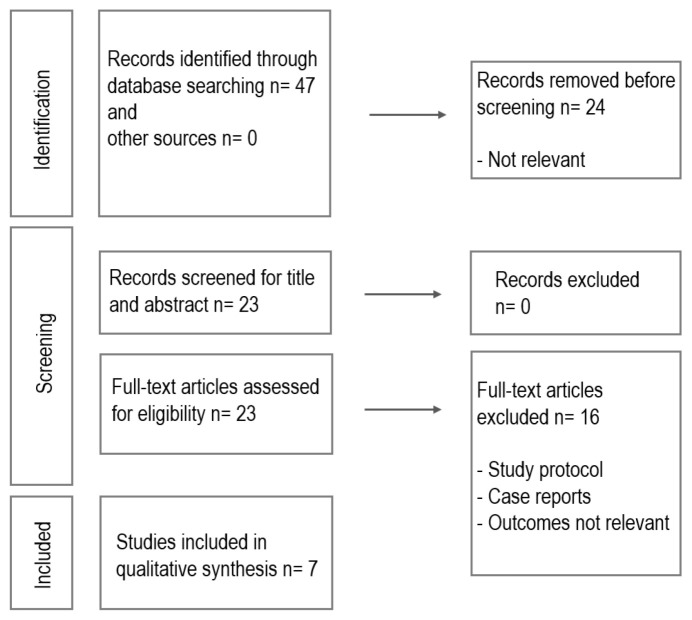
Diagram illustrating the search and article selection process.

Articles published in PubMed from inception up to December 2024 were searched, with no restriction on year of publication. Keywords with appropriate Boolean operators were used, using the search terms ‘preeclampsia’, ‘macular microcirculation’, ‘peripapillary capillary plexus’, ‘choriocapillaris’ and ‘optical coherence tomography angiography’. In addition, the reference lists of the retrieved articles were carefully reviewed for relevant literature. All studies that reported data on microcirculation characteristics such as the foveal avascular zone area, vascular density in all retinal capillary plexuses and the choriocapillaris were included. This identified 7 research studies [11,12,13,14,15,16,17] focusing on OCTA for evaluation of the microvasculature of chorioretinal layers at the macula and the peripapillary area in preeclampsia. The results of the search are summarized in Figure 1.

## 3. Results

### 3.1. Pathophysiology of Vasculature Changes in Pregnancy

Pregnancy induces a spectrum of progressive, physiological, cardiovascular, metabolic, hormonal, immunological and hematological adaptations to maintain an equilibrium for the developing fetus and preparation of maternal parturition [18,19]. The increased metabolic demands during pregnancy elicit a cascade of hemodynamic and blood flow distribution changes that may affect the systemic and ocular circulation. Despite the triggering of volume expansion mechanisms to increase circulating plasma volume and cardiac output, there is arterial underfilling, with 85% of blood volume residing within the venous circulation. In addition, estrogen, progesterone and renin–angiotensin levels are considerably increased in pregnancy. Both hemodynamic and hormonal changes lead to the upregulation of nitric oxide, contributing to a decrease in systemic vascular resistance and peripheral vasodilation [19,20,21,22,23,24,25,26,27,28,29].

Retinal blood flow autoregulation is achieved by the release of vasoactive mediators by the vascular endothelium and retinal tissue surrounding arterioles, leading to modifications to capillary vascular tone in response to changes in perfusion pressure or metabolic needs. This process may be compromised in pregnancy, leading to changes in ocular microcirculation including retinal capillary plexuses and choroidal vessels [19,20,21,22,23,24]. The synthesis of angiopoietic factors including angiopoietin-1,-2 and VEGF is also increased, contributing to the development and progression of retinal capillary dilation and perfusion changes. Previous experimental studies have indicated that the effect of these factors may also be modulated by growth hormones and insulin-like growth factors, both involved in fetal development. These pathophysiological changes may adversely affect vascular status, potentially leading to a variety of ocular disorders in pregnancy [19,20,21,22,23,24,25,26,27,28,29].

### 3.2. Ocular Manifestations in Preeclampsia

Hypertensive disorders of pregnancy, including preeclampsia, are common; however, ocular manifestations of clinical significance are rare. Preeclampsia may be triggered by abnormal placentation through trophoblast invasion in the uterus, diffuse endothelial cell dysfunction, the release of cytokines, vasoconstriction and platelet activation; however, the exact underlying etiological mechanism has not been fully elucidated [28,30]. Preeclampsia may lead to microcirculatory disruption and subsequent dysfunction of target organs [28,30], which may have a profound impact on the retinal and choroidal circulation, since these tissues require a constant blood flow to meet their high metabolic demands. The spectrum of ocular findings in preeclampsia ranges from mild physiological changes to severe, pathological, sight-threatening conditions requiring urgent therapeutic intervention. Visual symptoms may be reported in up to 25% of patients with severe pre-eclampsia, including blurred vision, photopsia, diplopia, visual field defects and, in severe cases, loss of sight [31,32,33,34,35].

The most common fundoscopic changes in preeclampsia are similar to those of hypertensive retinopathy. In the early stages, a variable degree of narrowing of the retinal arteriolar vessels may be present. Other findings include retinal hemorrhages, focal areas of ischemia of the retinal nerve fiber layer (cotton wool spots) and diffuse retinal edema or lipid exudation due to blood-retinal barrier dysfunction. In severe cases, increased intracranial pressure and ischemic optic neuropathy may lead to permanent visual deterioration [31,32,33,34,35].

Hypertensive choroidopathy mainly affects younger pregnant women with an acute elevation of blood pressure. Fundoscopy may reveal serous retinal detachment, Elschnig spots (infarctions of fibrinoid necrosis of the choriocapillaris that lead to atrophy in the overlying retinal pigment epithelium (RPE)) and Siegrist streaks (sclerosed choroidal vessels with overlying necrosis of the choriocapillaris). The presence of choroidopathy may indicate an aggressive form of hypertension, and prompt intervention is necessitated to prevent irreversible visual loss [31,32,33,34,35].

Exudative retinal detachment may occur in women with preeclampsia or after delivery, clinically presenting with a sudden loss of vision. It is hypothesized that uncontrolled hypertension may lead to choroidal ischemia with associated impaired RPE pump function subsequently resulting in an accumulation of subretinal fluid and a thickening of the choroid. Management involves the treatment of the underlying hypertension. Spontaneous resolution of the retinal detachment usually occurs within a few weeks, while visual prognosis is variable depending on the extent of RPE atrophy. Another possible cause of acute vision loss in women with preeclampsia may be cortical blindness. This is attributed to posterior cerebral circulation vasoconstriction, and visual recovery is usually achieved by 48–72 h postpartum [31,32,33,34,35].

The prompt management of preeclampsia requires medical control of hypertension, which is essential to attempt to reverse retinal changes and maximize visual outcomes. Delivery is the only curative intervention, so this is performed promptly when preeclampsia develops at or near term. Decisions about delivery when preeclampsia develops preterm are more complex, and the timing depends on the severity of preeclampsia [31,32,33,34,35].

### 3.3. OCTA Imaging Principles

OCTA imaging acquisition is based on the movement of erythrocytes; moving cells are distinguished from static tissue by sequentially acquiring B-scans from the same retinal location to depict high-resolution, three-dimensional maps of the retinal and choroidal capillary vasculature. OCTA generates enface images that provide structural and blood flow information on the posterior-segment vasculature, especially in the macula and peripapillary area, and depth resolution permits the acquisition of volumetric data and the segmentation of vascular layers. In this way, OCTA offers both a qualitative and quantitative assessment of the ocular microcirculation and is non-invasive without the need for intravenous dye [6].

OCTA can delineate retinal capillary layers in detail; these include the superficial capillary plexus (SCP), intermediate capillary plexus (ICP) and deep capillary plexus (DCP). Adequate imaging of the innermost part of the choroid, the choriocapillaris (CC), is also possible. By utilizing automated algorithms, OCTA enables the analysis of vessel density (VD), perfusion and foveal avascular zone (FAZ) parameters (area, perimeter, circularity index) of the macular area, and also provides information on the peripapillary capillary plexus. Several studies have described high accuracy and repeatability of OCTA metrics in normal eyes and in those with retinal vascular conditions [6].

However, OCTA has limitations that need to be considered. Firstly, it is not possible to distinguish a reduction in vascular diameter from the occlusion of vessels on OCTA, meaning that the exact cause of vascular alterations may not be identified [6]. Secondly, OCTA has a limited field of view of the posterior pole precluding the assessment of vasculature in the retinal periphery, as well as an inadequate visualization of the deep choroid. This may be of importance in conditions such as preeclampsia that affect the entire retinal and choroidal vasculature and have a multifactorial etiology including hypertension, hormonal and immunological factors. The use of widefield OCTA devices enabling the visualization of the chorioretinal vasculature over an extended field of the retina are now emerging in research and clinical practice. Imaging via widefield OCT and OCTA could provide a more global understanding of the retinal changes in preeclampsia in the future.

### 3.4. Imaging Chorioretinal Microcirculatory Changes in Normal Pregnancy

In undertaking retinal studies in pregnancy, there has been hesitancy about administering medications such as fluorescein dye for angiography [7,8,9,10]. Available studies using non-invasive imaging modalities such as OCT do provide evidence of subtle anatomical chorioretinal changes that are otherwise clinically undetectable, especially in the third trimester of pregnancy. Studies of healthy women demonstrated an increased total macular volume and retinal thickness on OCT scans at the end of pregnancy, presumably attributable to retinal capillary dilation arising from increased total blood volume and high levels of progesterone [36,37,38,39,40,41,42,43,44,45,46,47].

OCTA studies in healthy pregnant individuals have further characterized the normal compensatory microcirculatory changes that occur during this state. The perfusion density of the DCP has been found to be increased in pregnant women; however, this was not evident at the level of the SCP. A potential explanation for this difference might be due to physiological differences in hydrostatic pressure, and differing structure and function. In addition, the DCP is more vulnerable to ischemia than the SCP, which might be mitigated by the release of vasoactive mediators that lead to capillary adaptations to meet the metabolic demands of the tissue [7,10]. Changes in FAZ parameters have not been reported during pregnancy in healthy females, possibly because the fovea is relatively devoid of vessels, making small changes difficult to identify [8]. A number of studies have reported changes in choroidal thickness during pregnancy, but the results were variable; some studies found a thickening of the choroid, while others mentioned no difference from non-pregnant women. These differences could arise from reasons such as baseline characteristics of the study population, gestational age or imaging protocols. There is limited evidence on choriocapillaris (CC) blood flow, which has been evaluated by measuring CC flow voids (lack of vessels) on OCTA, and no difference between pregnant and non-pregnant women has been documented. These findings support the hypothesis that the choroid has a relatively even and constant homeostatic response that differs from the complex retinal autoregulatory process [41].

### 3.5. Chorioretinal Microcirculation and Blood Flow Alterations in Preeclampsia

The first report of evaluating retinal microcirculation characteristics in preeclampsia using OCTA was by Urfalioglu et al. in 2019 [11]. They conducted a prospective cross-sectional study including women with a confirmed diagnosis of non-severe preeclampsia (based on the criteria of the American College of Obstetricians and Gynecologists Association) (27 females), pregnant women without preeclampsia (26 females) and age-matched non-pregnant controls (25 females). The retinal capillary plexus and choriocapillaris within the macula and at the optic nerve head were imaged using a 6 × 6 mm^2^ scan centered on the fovea and the optic disk. Blood flow and vessel density of the superficial and deep retinal vascular plexus and choriocapillaris were analyzed in the parafoveal area (defined as a 3 mm circle centered on, but excluding, the fovea), and the optic nerve head in the peripapillary area. The authors demonstrated that (1) the retinal blood flow area and parafoveal vessel density in the superficial and deep capillary plexus were similar between pregnant (preeclamptic and healthy) women and non-pregnant controls (*p* > 0.05); and (2) choriocapillaris blood flow area was increased in pregnancy; however, it diminished in women with preeclampsia as compared to healthy pregnant females (*p* < 0.05). Similarly, optic nerve head blood flow was increased in healthy pregnancies compared to controls (*p* < 0.05); however, this was lower in women with preeclampsia as compared to those with a normal pregnancy, while preeclamptic and non-pregnant women had a similar blood flow (preeclamptic pregnant women had the lowest optic nerve head blood flow of all groups).

Ciloglu et al. evaluated the retinal microcirculation in pregnant women with preeclampsia (55 females), those with a normal pregnancy (43 females) and age-matched non-pregnant controls (38 females) in a prospective study [12]. All enrolled subjects were imaged by OCTA. Vascular density was calculated in the macular area over two regions of interest, the fovea and the parafovea (the latter defined as the ring-shaped area between a 0.3 and 1.5 mm radius from the center of the macula). The FAZ area was measured using a 3 × 3 mm^2^ scan and peripapillary images were acquired with a 4.5 × 4.5 mm^2^ scan centered on the optic disk. The peripapillary capillary vascular parameters were measured in a 1.00 mm wide elliptical annulus extending outward from the optic disk boundary in the radial peripapillary capillary zone. For the analyses, VD was automatically calculated for the disk and peripapillary area, whereas the authors analyzed the structural morphology of the peripapillary retinal nerve fiber layer (RNFL) thickness.

This study demonstrated changes in retinal microvascular structure on OCTA in women with preeclampsia, even without clinically evident retinal and optic disk pathology. The authors reported a decrease in both superficial and deep foveal vascular density and an increase in FAZ in pregnant women with preeclampsia compared to non-pregnant women. In addition, deep capillary plexus vascular parameters in the parafoveal area were decreased in preeclampsia compared to both the healthy pregnant and control women. Interestingly, there were no differences in microvasculature parameters between healthy pregnant and non-pregnant women. Concerning the peripapillary capillary plexus, this study demonstrated increased blood flow in pregnancies complicated by preeclampsia as compared to both normal pregnancies and women who were not pregnant. This finding correlated with increased peripapillary RNFL thickness, and the authors suggested that this may be due to the role of the peripapillary capillary plexus in maintaining RNFL metabolism and function.

Shim et al. evaluated OCTA in pregnancies complicated by preeclampsia and investigated any potential association with proteinuria and arterial blood pressure [13]. They conducted a retrospective study of 61 women with preeclampsia and analyzed FAZ and foveal and parafoveal VD in the superficial and deep capillary plexus. Blood flow parameters were quantified using the SS-OCTA imaging system. A 3 × 3 mm^2^ macula scan was taken; a 1 mm diameter circle centered on the fovea was defined as the foveal area, and an annular 3 mm area excluding the central 1 mm was defined as the paracentral area. Their study demonstrated that mean arterial pressure showed a significant linear relationship with protein–creatinine ratio, but there was no association of retinal microvasculature characteristics with these parameters (*p* > 0.05). The authors showed that choroidal thickness changes were correlated with the severity of preeclampsia.

Ozcan et al. performed a prospective case–control study [14] comparing three groups of patients: 50 with preeclampsia, 50 healthy pregnant and 50 non-pregnant women. Pregnant women with preeclampsia were evaluated at three different time points, including 3 h before delivery, 2–3 days after delivery and 6 weeks after delivery. All subjects underwent OCTA scanning of the macula and the optic disk. The macula was imaged via 3 × 3 mm^2^ fovea-centered scans and volumetric images were automatically segmented into en-face slabs of the superficial and deep capillary plexus, outer retina and choriocapillaris. The peripapillary area was analyzed using 4.5 × 4.5 mm^2^ scans centered on the optic disk and radial peripapillary capillary vessel density was acquired. The authors reported that deep parafoveal vessel density values were lower in women with preeclampsia compared to those with a healthy pregnancy; however, were widely restored in the postpartum period. The values between healthy pregnant and non-pregnant women were similar. Supporting these findings, Ciloglu et al. also reported that foveal and parafoveal vascular density in the superficial and deep capillary plexuses were lower in preeclampsia compared to healthy pregnant and non-pregnant women [12]. In addition, Ozcan et al. documented a significant reduction in superficial and deep foveal VD values in preeclampsia compared with their control groups (*p* < 0.05), while there was no difference in FAZ between all women (*p* > 0.05) [14]. A potential explanation of this discrepancy may be that the decrease in foveal VD results from diffuse capillary loss or hypoperfusion, rather than FAZ enlargement or remodeling.

Reduced blood flow was noted in the choriocapillaris layer in preeclampsia compared to healthy pregnancies. This finding was supported by Urfalioglu et al. and may be attributed to systemic and choroidal vasospasm in preeclampsia [11]. Furthermore, choroidal blood flow did not return to baseline values in women with preeclampsia in the postpartum period, although the underlying etiological mechanism remains unclear. Finally, radial peripapillary capillary density values were markedly lower in preeclampsia than in healthy pregnancies and in the non-pregnant state, though they increased in the postpartum period.

Fayed et al. [15] undertook a prospective, observational, cross-sectional, comparative analysis of the retinal microcirculation in the third trimester of pregnancy, comparing 15 women with a healthy pregnancy, 15 women with a pregnancy and systemic hypertension and 15 with preeclampsia. The authors explored choriocapillaris blood flow in the parafoveal area by calculating the choroidal adjusted flow index. OCTA imaging with split-spectrum amplitude-decorrelation angiography software was performed. The scan area included 3 × 3 mm^2^ and 6 × 6 mm^2^ scans centered on the fovea, and the images were automatically segmented on the choriocapillaris. The authors demonstrated significantly lower parafoveal choroidal blood flow in the third trimester in pregnant women with preeclampsia compared to those with a healthy pregnancy and pregnancy with systemic hypertension. In addition, they highlighted that this significantly lower choroidal perfusion in the eyes of women with preeclampsia may suggest that a subtle threshold of choroidal ischemia exists, above which the level of tissue deoxygenation may not be tolerated by the retinal pigment epithelium, paving the way for the various retinal and choroidal changes seen in severe cases of preeclampsia.

Hoel et al. aimed to assess the structural and functional properties of the retinal arterioles, venules and capillaries in women with hypertensive disorders of pregnancy (gestational hypertension, in particular preeclampsia) [16]. The retinal microvasculature was assessed by vessel caliber measurements, retinal oximetry and OCTA at 3 years postpartum in 27 women with previous hypertensive disorders of pregnancy and 23 healthy controls. OCTA imaging was performed using a 6 × 6 mm^2^ scan centered on the fovea. The macular microcirculation was evaluated by assessing the vessel density, vessel skeleton density and vessel diameter index in the perifovea area (1–6 mm annular area around the fovea) of the superficial capillary plexus. To ensure the validity of the analyses, the deep capillary plexus was excluded due to shadowing by the superficial capillary plexus, while the inner 1 mm circle was also excluded, since it involved the FAZ, which could exhibit individual heterogeneity. The authors’ hypothesis was that women with previous hypertensive disorders of pregnancy would have a reduced retinal microvascular density compared to women with uncomplicated pregnancies. Interestingly, they showed that all macular vascular density metrics were comparable between the groups (all *p* > 0.05) at 3 years postpartum, suggesting the reversibility of any microvascular changes.

Pota et al. undertook a single-center study [17] comparing 27 pregnant women with preeclampsia, 30 healthy pregnant and 30 non-pregnant women. All participants underwent imaging with SS-OCTA, and fovea-centered 3 × 3 mm^2^ scans were used for microvasculature analysis. An area of 1 mm in diameter centered on the fovea was considered the central area, and the 3 mm diameter area surrounding this area was considered the parafoveal area. Automated VD measurements were performed in the foveal and parafoveal areas (superior, inferior, temporal and nasal quadrants) to analyze the retinal layers and choriocapillaris, and the FAZ area was measured manually. This study reported lower values of the FAZ area and foveal VD in the superficial and deep capillary plexus in women with preeclampsia than in those with a healthy pregnancy; however, there was an enlargement of the FAZ area in both healthy pregnant women and those with preeclampsia as compared to non-pregnant women. In addition, the authors demonstrated that the VD in the parafoveal area was higher in pregnant women with preeclampsia than in non-pregnant women; the latter were similar to those with a healthy pregnancy. They also demonstrated a significant increase in the choriocapillaris VD in both women with preeclampsia and a healthy pregnancy compared to those who were not pregnant (*p* < 0.01).

**Figure 2:**
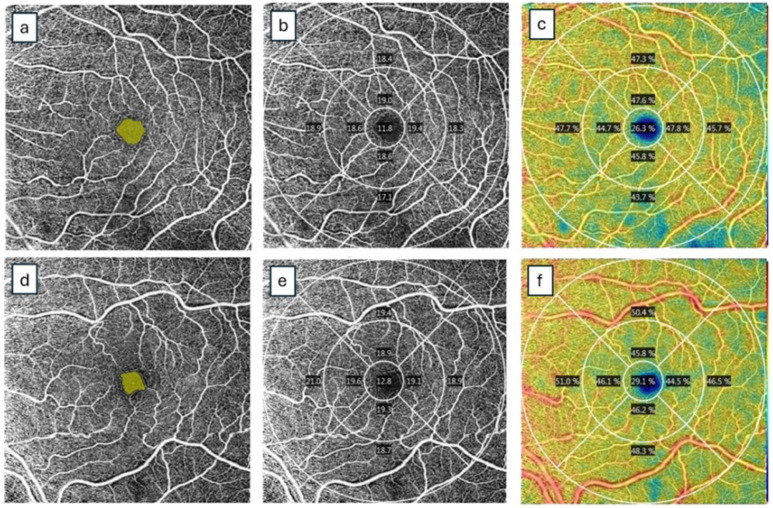
Representative macular OCT angiography images from a woman with preeclampsia (without fundoscopic findings of retinopathy) and a woman with a healthy pregnancy.

A summary of the research studies’ characteristics and results is shown in Table 1. Representative macular OCTA images from a woman with preeclampsia and a woman with a healthy pregnancy are illustrated in Figure 2.

Macular scans (6 × 6 mm^2^) of the left eye are shown in both patients. En-face images of the retinal microvasculature were generated for the superficial capillary plexus (SCP) based on an automated layer segmentation performed by AngioPlex^®^ OCT Angiography system software (Zeiss Cirrus HD-5000 Spectral-Domain OCT with AngioPlex OCTA, Jena, Germany). The foveal avascular zone (FAZ) area (mm^2^), vessel density (VD) (mm/mm^2^) and perfusion density (PD) (%) in the SCP were automatically calculated. The FAZ area is delineated by the yellow circle (images a, d). The ETDRS macular grid chart delineates subfields of the foveal (central), parafoveal (inner ring) and perifoveal (outer ring) areas, indicating the values of VD (images b, e) and PD (images c, f) in each quadrant. In the patient with preeclampsia, the FAZ SCP area was 0.29 mm^2^, perimeter 2.13 mm and circularity 0.81 (a). The SCP mean vessel density (mm/mm^2^) values in the ETDRS grid were 11.8 in the central area, 18.9 in the inner ring and 18.1 in the outer ring. The full area was 18.1 (b). The average perfusion density values (%) in the SCP were 26.3 in the central area, 46.5 in the inner ring, 46.1 in the outer ring and 45.6 in the full area (c). In the patient with a healthy pregnancy, the FAZ SCP area was 0.21 mm^2^, perimeter 1.94 mm and circularity 0.70 (d). The SCP mean vessel density (mm/mm^2^) values in the ETDRS grid were 12.8 in the central area, 19.2 in the inner ring and 19.4 in the outer ring. The full area was 19.1 (e). The average perfusion density values (%) in the SCP were 29.1 in the central area, 45.6 in the inner ring, 49.1 in the outer ring and 47.7 in the full area (f).

## 4. Discussion

Pregnancy is associated with elevated cardiac output, increased intravascular volume and peripheral vascular dilation, which increases systemic blood flow to organs including the eye. In preeclampsia, complex blood flow dynamics might lead to the dysregulation of the chorioretinal vascular system [19,20,21,22,23,24,25,26,27,28,29] via increased perfusion pressure, causing a vasogenic edema resulting in tissue ischemia.

In this review, we assessed the current evidence on OCTA for assessing retinal microcirculation parameters in preeclampsia, and report variations across the published literature. Changes in the FAZ area and macular VD were detected in some studies and not in others and, if present, there were demonstrated changes in different chorioretinal layers. Overall, reduced blood flow in the retinal microvasculature and vascular density were reported in two studies [12,14], and both indicated that the DCP was more severely affected compared to the SCP in preeclampsia. In two further studies [11,16], SCP and DCP metrics did not significantly differ in women with preeclampsia as compared to healthy pregnant and non-pregnant women, whilst one study [17] demonstrated variability between macular regions.

It is hypothesized that preeclampsia might affect the deep more than the superficial capillary layers. Potential explanations for this discrepancy might be differing distance from larger arterioles, the proximity of the DCP to the outer retina and its high metabolic demand and the complex vascular anatomic architecture. This finding is in line with previous reports describing different patterns of flow resistance and perfusion in different retinal capillary plexuses [19,21,24,29]. Furthermore, former studies [6,29] have provided evidence that systemic vasospasm may have more of an adverse effect on the deep capillary plexus of the retina, which is associated with high metabolic activity that might make it more vulnerable to ischemia. Numerous other parameters such as blood volume, an increase in cardiac output, a decrease in peripheral vascular resistance, vasodilation, hormonal changes and the duration of hypertension might have an impact on these outcomes in pregnancy.

Concerning FAZ area, there was no consensus in the findings of the studies reported. Two studies [12,17] reported an enlargement of the FAZ area in pregnancy (healthy or preeclampsia), along with diminished foveal vessel density. However, a further study [14] did not find any FAZ changes in correlation to diminished foveal VD. A potential explanation of this discrepancy between studies may be that the decrease in foveal VD results from diffuse capillary loss or hypoperfusion, rather than FAZ enlargement or remodeling.

An assessment of choriocapillaris blood flow revealed consistent results between three studies [11,14,15], demonstrating increased blood flow in pregnancy compared to the non-pregnant state. In contrast to the SCP and DCP, the choriocapillaris has no autoregulation and remains under the control of the autonomic nervous system. Therefore, the higher ocular perfusion pressure caused by increased systemic blood flow could explain this increase in choriocapillaris blood flow during pregnancy. However, the increase seen in women with preeclampsia was lower than in healthy pregnancies. A potential explanation of this finding might be that there is choroidal vascular spasm and decreased blood flow secondary to the generalized systemic vasospasm in preeclampsia. Interestingly, only one study indicated similar values in both healthy and preeclamptic pregnant women [17].

Finally, three of the studies [11,12,14] evaluated peripapillary blood flow, providing inconsistent outcomes. Ciloglu et al. showed that peripapillary capillary density was significantly higher in women with preeclampsia than in the control group and showed a positive correlation with RNFL values [12]. However, Ozcan et al. found a markedly lower peripapillary capillary density in women with preeclampsia compared to controls, and suggested that the association with RNFL thickness may not be valid for preeclamptic women (despite being potentially present in healthy subjects) since the increased RNFL thickness may result from fluid retention, and peripapillary capillary density values may decrease because of increased vascular resistance [14]. In addition, the radial peripapillary capillary network is more sensitive to reduced blood flow because it is straighter and longer than other retinal capillaries and connects large arteries and veins directly without anastomosis.

Possible explanations for the overall discrepancies between the study results could include the heterogeneity of the baseline characteristics of the participants (e.g., age, ethnicity, gestational age), the severity of preeclampsia (and the potentially variable definitions used), the use of different OCTA machines, different regions of the macular and peripapillary areas that were assessed and the number of patients examined in each study. In some cases, the characteristics of the pregnant women may not have been reported, leading to possible bias. For example, it may be that the women enrolled into studies where no retinal microvascular changes (SCP and DCP) were detected had a milder systemic disease, and retinal autoregulatory mechanisms could still compensate for these changes. In addition, the intrinsic limitations of OCTA technology, the interpretation of chorioretinal vasculature imaging and the interference of projection artifacts (especially in the assessment of the choriocapillaris) may contribute to these disparities. This review therefore demonstrates the need for further research that requires a more transparent documentation of the participants’ characteristics, a standardization of acquisition protocols and larger-scale and longitudinal studies to more reliably assess retinal microvascular parameters in pregnancy and preeclampsia.

Visual symptoms are not uncommon in pregnancy. However, they are sometimes not prioritized in the setting of acute severe hypertension where urgent delivery is planned to address potential fetal compromise. In this scenario, ophthalmic assessment in women with visual symptoms may be restricted by the requirements for frequent blood pressure and fetal monitoring. At a minimum, ocular evaluation could include bedside testing of visual acuity and dilated fundus examination. As soon as is practicable after the diagnosis of preeclampsia, or after delivery, comprehensive retinal imaging is advisable in visually symptomatic women, which could encompass widefield fundus images, OCT of the macula and optic disk and in some cases a formal visual field assessment. OCTA may provide useful information on the severity of chorioretinal vascular involvement, especially in the context of otherwise unexplained visual symptoms. Additionally, it could offer the potential to detect microvasculature changes prior to clinically evident ocular manifestations in early stages of preeclampsia. Future research with large prospective multicenter studies involving multiethnic populations could allow us to stratify the outcomes of microvasculature changes by preeclampsia severity. If more evidence emerged regarding the ability of OCTA to distinguish between isolated hypertension in pregnancy and the more widespread endothelial dysfunction associated with preeclampsia, there would be a role for early OCTA imaging in development for suspected preeclampsia as a marker to support prompt and timely interventions in these women. In that respect, OCTA could guide decision making and contribute to optimizing visual outcomes in clinical practice.

## 5. Conclusions

The retinal microcirculation may provide insights into systemic vascular status, and therefore may reflect vascular pathology in women with pregnancies complicated by preeclampsia. An analysis of the chorioretinal capillary plexus utilizing OCTA may potentially characterize ocular vascular manifestations in this disorder. This narrative review article summarizes OCTA parameters that may be utilized for the assessment of the pathophysiology of chorioretinal vascular manifestations in preeclampsia such as FAZ, SCP and DCP vessel density, choriocapillaris and peripapillary blood flow. The current evidence demonstrates quantitative vascular flow alterations in the macula and the peripapillary area, although their characterization in preeclampsia needs to be further elucidated since the conclusions of studies reviewed were not always consistent, likely as a result of their heterogeneity in multiple factors. Further research including longitudinal studies are warranted to clarify the role of OCTA characteristics in preeclampsia and determine the accuracy and long-term usefulness of chorioretinal vascular metrics in clinical practice. 

## Figures and Tables

**Table 1:** Summary of research studies’ characteristics and results.

Study	Design	Study Population			OCTA Device	Scan Area (mm²)		OCTA Characteristics						Study Outcomes
		Preeclampsia	Healthy Pregnancy	Non-Pregnancy		Macula	Optic Nerve	FAZ		VD Macula			VD Optic Nerve	
								SCP	DCP	SCP	DCP	CC		
Urfalioglu et al., 2019 [11]	Prospective, cross-sectional	27	26	25	AngioVue, Optovue, RTVue-XR, Fremont, CA, USA	6 × 6	6 × 6	-	-	+	+	+	+	Retinal microcirculation maintained consistent flow; choriocapillaris and optic nerve head vasculature were attenuated in preeclampsia
Ciloglu et al., 2019 [12]	Prospective	55	43	38	AngioVue, OptoVue, RTVue-XR, Fremont, CA, USA	3 × 3	4.5 × 4.5	+	+	+	+	-	+	Retinal microvasculature structure changes in preeclampsia, even without confirmed retinal and optic disk pathology
Shim et al., 2021 [13]	Retrospective	61	-	-	DRI OCT, Triton, Topcon, Tokyo, Japan	3 × 3	-	+	+	+	+	-	-	Arterial pressure and protein–creatinine ratio indicated no association with retinal microvasculature
Ozcan et al., 2022 [14]	Prospective, cross-sectional	50	50	50	AngioVue, OptoVue, RTVue-XR, Fremont, CA, USA	3 × 3	4.5 × 4.5	+	+	+	+	+	+	There was a decreased macular DCP VD in preeclampsia, which showed improvement after delivery
Fayed et al., 2023 [15]	Prospective, observational, cross-sectional, comparative	15	15	-	AngioVue, OptoVue, RTVue-XR, Fremont, CA, USA	3 × 3, 6 × 6	-	-	-	-	-	+	-	There was low choroidal blood flow in preeclampsia compared to pregnancy with systemic hypertension and healthy pregnancy
Hoel et al., 2024 [16]	Pilot study	27	-	23	Zeiss PLEX Elite 9000, Carl Zeiss, Meditec Inc, Dublin, CA, USA	6 × 6	-	-	-	+	-	-	-	Microvasculature structural alterations were not biomarkers for cardiovascular disorders in healthy women 3 years postpartum with hypertensive disorders in pregnancy
Pota et al., 2024 [17]	Single center	27	30	30	DRI OCT, Triton, Topcon, Tokyo, Japan	3 × 3	-	+	+	+	+	+	-	In preeclampsia, a decreased central VD, increased FAZ with increased systolic blood pressure and decreased choroidalthickness could contribute to monitoring

Abbreviations: VD, vessel density; FAZ, foveal avascular zone; SCP, superficial capillary plexus; DCP, deep capillary plexus; CC, choriocapillaris; +, reported parameter; -, non-reported parameter.
